# Prediction-Guided Biotransformation of *p*-Hydroxyphenethyl Anisate into a Novel 3',4'-Dihydroxyphenethyl Anisate with Potent Anti-Inflammatory, Anti-Melanoma, and Antioxidant Activities

**DOI:** 10.4014/jmb.2601.01040

**Published:** 2026-05-11

**Authors:** Chien-Yu Wu, Hsiou-Yu Ding, Huei-Ju Ting, Tzi-Yuan Wang, Tzu-Hsuan Li, Jou-Yi Chen, Hui-Ling Huang, Jiumn-Yih Wu, Te-Sheng Chang

**Affiliations:** 1 Department of Biological Sciences and Technology, National University of Tainan, Tainan 700301, Taiwan; 2 The integrative Evolutionary Galliform Genomics (iEGG) and Animal Biotechnology Center, National Chung Hsing University, Taichung 402202, Taiwan; 3 Department of Cosmetic Science, Chia Nan University of Pharmacy and Science, Tainan 717301, Taiwan; 4 Biodiversity Research Center, Academia Sinica, Taipei 115201, Taiwan; 5 Department of Food Science, National Quemoy University, Kinmen County 892009, Taiwan

**Keywords:** Anti-inflammation, Anti-melanoma, Antioxidant, Hydroxylation, Predicted data mining approach, Biotransformation

## Abstract

Discovering novel bioactive compounds is a fundamental goal in scientific research. To this end, our study employed a predicted data mining approach (PDMA) to efficiently screen for biotransformable precursors capable of yielding new bioactive derivatives. Using the PDMA strategy, we identified *p*-hydroxyphenethyl anisate (HP) as a substrate and employed *Bacillus megaterium* tyrosinase (*Bm*TYR) to catalyze the hydroxylation of HP. The results demonstrated that HP was successfully converted into a novel *ortho*-dihydroxyphenyl compound, 3',4'-dihydroxyphenethyl anisate. This biotransformation yielded a product with significantly enhanced therapeutic potential. Notably, the product showed a potent 11-fold increase in anti-inflammatory activity compared to the precursor. Mechanistically, 3',4'-dihydroxyphenethyl anisate effectively mitigated the hyperimmune response in lipopolysaccharide-stimulated RAW 264.7 macrophages by suppressing the gene expression of pro-inflammatory mediators, including tumor necrosis factor-alpha, interleukin-1 beta, interleukin 6, inducible nitric oxide synthase (iNOS), and cyclooxygenase-2. Notably, Western blot analysis confirmed that this inhibitory effect was sustained at the protein level, where 3',4'-dihydroxyphenethyl anisate induced a significant downregulation of iNOS expression. Furthermore, the hydroxylated product showed preliminary antioxidant capacity (absent in the parent compound) and observable anti-melanoma activity. This study validates PDMA as an effective strategy for generating novel and high-value bioactive molecules via biotransformation. Our newly produced catechol is a promising candidate for future pharmacological applications.

## Introduction

The ongoing need for novel therapeutics to address a wide range of human diseases has fueled sustained research efforts toward the discovery of new bioactive compounds. However, conventional synthesis of new molecules in organic chemistry and the pharmaceutical sector often relies on chemical catalysis, which is associated with significant drawbacks such as high operational costs and the generation of environmentally detrimental waste [1-3]. Recent advances in biochemistry have revealed a vast repertoire of biocatalytic enzymes that enable the sustainable synthesis of novel products under precisely controlled and mild reaction conditions [4, 5]. These enzymes can catalyze a diverse array of transformations, including hydroxylation, glycosylation, hydrogenation, dehydrogenation, hydrolysis, *O*-methylation, and *O*-acetylation [6-10]. Such biocatalytic methods are powerful tools for modifying existing molecular scaffolds with exceptional enantioselectivity and regioselectivity, thereby accelerating the identification of new chemical entities. These biocatalytic approaches aim to substantially decrease dependence on nonselective, hazardous industrial reagents and minimize waste, ultimately facilitating the synthesis of value-added products in an environmentally responsible manner [1-3, 10].

Nevertheless, the enzymatic conversion of known compounds into novel bioactive molecules often requires laborious and cost-intensive experimental optimization [11, 12]. To overcome these limitations, our group developed the predicted data mining approach (PDMA), an expedited methodology for identifying new biotransformation products from known enzymes and substrates; this approach has been proven effective in multiple applications [11]. This approach was initially applied to the hydroxylation of phenolic compounds by *Bacillus megaterium* tyrosinase (*Bm*TYR) to synthesize novel catechol derivatives [11]. The utilization of PDMA, however, extends to diverse biocatalytic reactions, as demonstrated by our successful application of tyrosinase for the *ortho*-hydroxylation of numerous flavonoids and stilbenoids [12-16]. Furthermore, our recent work has shown that this biotransformation approach extends beyond natural products to include synthetic molecules, such as the drug isoxsuprine and the chemical dye 4-[(2',4'-dinitrophenyl)amino]-phenol [17, 18].

Hydroxylation, the enzymatic conversion of a carbon–hydrogen (C–H) bond to a carbon–hydroxyl (C–OH) bond, is a ubiquitous biochemical reaction [19]. This oxidative transformation plays a fundamental role in the metabolism and functionalization of diverse organic substrates, including pharmaceuticals, plant-derived natural products, and environmental xenobiotics [19, 20]. For flavonoids specifically, hydroxylation has been reported to potentiate their antioxidant capacity [11, 14, 21], augment their inhibitory effects against α-glucosidase and α-amylase [11, 22, 23], and enhance their anti-inflammatory properties [17, 18, 24].

Using the PDMA for database screening, we identified *p*-hydroxyphenethyl anisate (HP) as a promising candidate. This compound can be hydroxylated by *Bm*TYR to yield novel compounds. HP is one of the major constituents isolated from the traditional Chinese herb Qiang huo (*Notopterygii rhizoma et Radix*). [25-27]. Qiang huo is a historic Chinese medicinal herb, traditionally prescribed for inflammatory disorders, such as rheumatoid arthritis [26-28]. It has also been applied as an analgesic for headaches and a diaphoretic to induce sweating for treating the common cold [26-28]. Modern pharmacological studies have demonstrated its anti-inflammatory, antibacterial, antioxidant, antiarrhythmic, anticancer, antipyretic, and analgesic activities [27, 28]. Our recent research has also demonstrated that HP possesses potent anti-melanogenic activity [29]. However, to date, HP has not yet been investigated as a substrate for biotransformation to synthesize new compounds.

In this study, HP was selected as a substrate for biotransformation through PDMA screening. We predicted that *Bm*TYR could *ortho*-hydroxylate its phenolic structure to form a novel catechol derivative. We executed this biotransformation and then purified and structurally characterized the novel product. Subsequently, we assessed its bioactivities, hypothesizing that the *ortho*-hydroxylation would enhance its therapeutic potential. We assessed its antioxidant capacity (2,2-Diphenyl-1-picrylhydrazyl (DPPH) assay) and anti-melanoma cytotoxicity, finding that both were significantly enhanced compared with those of the precursor HP. We also investigated its anti-inflammatory activity in cellular models and its modulatory effect on pro-inflammatory gene expression at the molecular level.

## Materials and Methods

### Chemicals, Enzymes, and Cell Lines

HP was purchased from Baoji Herbest Bio-Tech (Xi-An, China). *Bm*TYR with a specific activity of 4.84 U/mg was prepared as previously described [15]. All additional reagents and solvents were purchased from commercial suppliers. The murine macrophage cell line RAW 264.7 (BCRC 60001) and murine B16 melanoma cells (BCRC60030) were obtained from the Food Industry Research and Development Institute’s Bioresources Collection and Research Center (BCRC, Hsinchu, Taiwan). The cells were cultured according to BCRC's guidelines.

### Predicted Data Mining Approach (PDMA)

The strategy for precursor selection was adapted from our previous work [11]. Candidate compounds were evaluated using three predefined criteria: (1) the presence of a phenyl group structurally analogous to tyrosine for recognition and catalysis by *Bm*TYR; (2) commercial availability in Gram-scale quantities to ensure sufficient material for product synthesis and subsequent analysis; and (3) the novelty of the predicted biotransformation product. To meet the first two criteria, commercial chemical catalogs were surveyed. To assess novelty (criterion 3), the putative *ortho*-hydroxylated product of each promising candidate was computationally generated using the Reaxys program (RELX, UK). The resulting structures were then queried against both the SciFinder^®^, and PubChem databases to confirm they were not previously reported. Precursors predicted to yield novel derivatives were subsequently selected for experimental validation through *Bm*TYR biotransformation.

In the present study, a customized commercial list with 430 natural compounds was obtained from Baoji Herbest Bio‐Tech (Xi‐An). From the 430 natural compounds, 41 compounds (9.5% of the total) were selected as the candidates of *Bm*TYR substrates based on the chemical phenolic structure. All the predicted products, *ortho*-dihydroxyl derivatives of the 41 compounds, were drawn and searched in SciFinder^®^ and PubChem databases. One product, 3',4'-dihydroxyphenethyl anisate, derived from hydroxylation of HP was found as a new compound and did not exist in the databases. Accordingly, HP was purchased and subjected to the biotransformation by *Bm*TYR.

### Determination of Tyrosinase Activity

Tyrosinase activity of the purified *Bm*TYR was determined following a previously described method [14]. The assay mixture consisted of 0.1 mL of 2.5 mM L-DOPA prepared in phosphate buffer (PB, pH 6.8) and *Bm*TYR at a final concentration of 108 μg/mL. The reaction was incubated at 25°C for 2 min. Dopachrome production (ε = 3600 M^-1^ cm^-1^) was monitored at 475 nm using a microplate reader (Sunrise, Tecan, Switzerland). One unit of *Bm*TYR activity was defined as the amount of enzyme required to produce 1 μmol of dopachrome per minute under the specified assay conditions. The purified recombinant *Bm*TYR exhibited a specific L-DOPA oxidation activity of 4.84 U/mg.

### Biotransformation by *Bm*TYR

The biotransformation reaction was performed based on a previously described method with minor modifications [16]. Briefly, reactions were conducted in a 100 μL total volume containing 500 mM borate buffer (pH 9.0), 10 mM ascorbic acid, and 1 mg/mL substrate. The substrate was added from a 20 mg/mL stock in dimethyl sulfoxide (DMSO), resulting in a final DMSO concentration of 5%. The reaction was initiated by the addition of *Bm*TYR to a final concentration of 108 μg/mL. The mixture was then incubated for 1.5 h at 50°C with agitation at 200 rpm. To quench the reaction, 20 μL of 1 M HCl was added, followed by 120 μL of methanol (MeOH). The samples were then analyzed using high-performance liquid chromatography (HPLC).

### HPLC Analysis and Purification

HPLC analysis was performed according to a previously established method [30]. The chromatographic system consisted of an Agilent 1100 series instrument (Agilent, USA) equipped with a Waters 600 gradient pump (Waters, USA) and controlled by Chromatography Data Station software (Scientific Information Service Co., Ltd., Taiwan). Separations were achieved on a Sharpsil H-C18 column (5 μm, 4.6 i.d. × 250 mm; Sharpsil, China) at a flow rate of 1 mL/min. The mobile phase was composed of 1% acetic acid in water (solvent A) and methanol (solvent B). The gradient elution profile was as follows: 20% B at 0 min, linear increase to 50% B at 20 min, isocratic hold at 50% B until 25 min, return to 20% B from 25 to 28 min, and hold at 20% B until 35 min. For each analysis, a 10 μL sample was injected, and the eluate was monitored at a wavelength of 254 nm.

For structural characterization, the biotransformation was scaled up to a total volume of 20 mL. After the reaction, the metabolites were purified by preparative HPLC using a Young Lin YL9100 system (YL Instrument, Republic of Korea). Fractions corresponding to the product peak were collected, concentrated in vacuo using a rotary evaporator (Rotavapor R-100, Büchi, Japan), and lyophilized.

### Structural Characterization (NMR and Mass Spectrometry)

The structure of the purified compound was elucidated using mass spectrometry and nuclear magnetic resonance (NMR) spectroscopy. The mass spectra were acquired on an AB Sciex QTRAP 5500 mass spectrometer (SCIEX, USA) equipped with an electrospray ionization (ESI) source. Data were collected in the positive ion mode over a mass range of *m/z* 100–1150 using single-ion recording. The capillary voltage was set between 4.5 and 5.5 kV, and the desolvation gas pressure was 20 psi. Data were processed using Analyst 1.5 software.

Full mass and mass-mass scan data were acquired over a mass range of 100 to 1,150 *m/z* in positive ion mode. For the NMR analysis, a 5-mm-diameter NMR tube containing 5 mg of the purified product compound dissolved in 0.5 mL of DMSO-*d6* was prepared. Subsequently, ^1^H- and ^13^C-NMR, distortionless enhancement polarization transfer (DEPT), heteronuclear single quantum coherence (HSQC), heteronuclear multiple-bond connectivity (HMBC), correlation spectroscopy (COSY), and nuclear Overhauser effect spectroscopy (NOESY) were recorded on a high-resolution Bruker AV-700 NMR spectrometer (Bruker Co., USA) at ambient temperature. Standard pulse sequences and parameters were used for the NMR experiments, and all chemical shifts were reported in parts per million (ppm, δ).

### In silico Pharmacokinetic and Toxicity Analysis

The simplified molecular input line entry system notation of 3',4'-dihydroxyphenethyl anisate obtained from Reaxys^®^ was entered into the pkCSM (https://biosig.lab.uq.edu.au/pkcsm/ (accessed October, 2025)) [31] and VenomPred 2.0 (https://www.mmvsl.it/wp/venompred2/ (accessed October, 2025)) [32] to assess its pharmacokinetic and toxicity profiles.

### Determination of Anti-Melanoma Activity

Anti-melanoma activity was determined using an MTT [3-(4,5-dimethylthiazol-2-yl)-2,5-diphenyltetrazolium bromide] cytotoxicity assay, adapted from a previous protocol [33]. Melanoma cells were maintained in Dulbecco's modified Eagle's medium (DMEM) (10% fetal bovine serum [FBS]) at 37°C in a 5% CO_2_ incubator. The cells were seeded and incubated for 24 h prior to a 48-h treatment with the test compounds. Celastrol served as a positive control to evaluate the comparative anti-melanoma efficacy of 3',4'-dihydroxyphenethyl anisate [47]. Subsequently, cell viability was quantified by replacing the medium with MTT (1 mg/mL in phosphate buffered saline [PBS]) and incubating for 2 h. The resulting formazan was solubilized in DMSO, and optical density (OD) was measured at 570 nm. The percentage of cell survival was calculated relative to the untreated vehicle control using the following equation: cell survival (%) = [OD_570_ treated) / (OD_570_ control)] × 100%. The IC_50_ value, representing the compound concentration that resulted in 50% cell survival, was determined from the dose–response curves.

### Measurement of Antioxidant Activity

The antioxidant capacity of the compounds was assessed via a DPPH radical scavenging assay, following a previously published protocol [15] with minor modifications. Test samples and the positive control, ascorbic acid, were prepared as stock solutions in DMSO. The assay was performed by adding the sample to a 1 mM solution of DPPH in methanol to achieve a final reaction volume of 100 μL. Following a 15 min incubation period, the decrease in absorbance was monitored at 520 nm with a Tecan Sunrise microplate reader. The radical scavenging activity was calculated as the percentage reduction in absorbance relative to a control containing only DMSO. The results were expressed as the IC_50_ value, representing the concentration of the test compound that inhibited 50% of the DPPH free radicals.

### Evaluation of Anti-Inflammatory Activity

The anti-inflammatory potential of the compounds was assessed by measuring their ability to inhibit nitric oxide (NO) production in LPS-stimulated RAW 264.7 macrophages, as previously described [33]. Cells were cultured in DMEM supplemented with 10% FBS and antibiotics at 37°C and 5% CO_2_. For the assay, cells were seeded at 5 × 10^5^ cells/well in 24-well plates. After 12 h, they were pre-treated with compounds for 1 h before being challenged with 6.25 ng/mL LPS for 24 h. 8-Hydroxydaidzein (8-OHDe) served as a positive control to evaluate the comparative anti-inflammatory efficacy of compounds [17]. NO concentration in the culture supernatant was quantified using the Griess reagent, with absorbance measured at 540 nm on a Tecan Sunrise microplate reader. Immediately following this, the cytotoxicity of the compounds was evaluated in the same wells via an MTT assay. After a 2 h incubation with MTT (1 mg/mL in PBS), the resulting formazan was solubilized in DMSO, and absorbance was measured at 570 nm. All treatments were performed in triplicate. The IC_50_ for NO inhibition was calculated, and cell viability was expressed as a percentage relative to the vehicle-treated control.

### ELISA Assays

RAW 264.7 cells were pre-treated with test compounds for 1 h prior to stimulation with 6.25 ng/mL LPS for 24 h. 8-OHDe served as a positive control to evaluate the comparative anti-inflammatory efficacy of test compounds [17]. Cell supernatants were then collected, and the levels of interleukin 6 (IL-6) were determined with the Uncoated Mouse IL-6 ELISA Kit (Elabscience, USA).

### Total RNA Isolation and Quantitative PCR (qPCR)

The experimental protocols for RNA isolation and gene expression analysis were adapted from previous studies [17]. Briefly, RAW 264.7 cells were seeded into six-well plates (3 × 10^6^ cells/well) and incubated for 24 h at 37°C in a 5% CO_2_ atmosphere. The cells were subsequently pre-treated for 1 h with 3',4'-dihydroxyphenethyl anisate (10 or 40 μM) prior to stimulation with 6.25 ng/mL LPS for 6 or 24 h. Total RNA was extracted from the cells using Trizol reagent (Thermo Fisher Scientific, USA) according to the manufacturer’s instructions, which involved chloroform extraction and isopropanol precipitation (all centrifugation steps at 12,000 × *g*, 4°C). RNA quantity and purity (A260/A280) and integrity (gel electrophoresis) were verified. Following isolation, total RNA was treated with DNase I (Promega, USA) to remove genomic DNA. First-strand cDNA was then synthesized using the iScript™ cDNA Synthesis Kit (Bio-Rad, USA). qPCR was performed on a StepOnePlus™ Real-Time PCR System (Applied Biosystems, Thermo Fisher Scientific) using iTaq Universal SYBR Green Supermix (Bio-Rad, USA). Each 20 μL reaction contained 15 ng of cDNA template and 400 nM of forward and reverse primers. Gene expression levels were normalized to GAPDH as the internal control. The sequences of the primers used were as follows: cyclooxygenase-2 (COX-2) forward: GCGACATACTCAAGCAGGAGCA, reverse: AGTGGTAACCGCTCAGGTGTTG; interleukin-1 beta (IL-1β) forward: CCTGGGCTGTCCTGATGAGAG, reverse: TCCACGGGAAAGACACAGGTA; IL-6 forward: AAGTGCATCATCGTTGTTCATACA, reverse: GAGGATACCACTCCCAACAGACC; inducible nitric oxide synthase (iNOS) forward: ATGGAGCATCCCAAGTACGAG, reverse: CTCCAGGATGTTGTAGCGCTG ; tumor necrosis factor-alpha (TNF-α) forward: ACTGTGGGCCTCTCATGC, reverse: TTGCAGAACT CAGGAATGG; and GAPDH forward: CAATGTGTCCGTCGTGGATCT, reverse: GTCCTCAGTGTAGCCCAAGATG.

### Western Blot Analysis

Cells were seeded at a density of 3 × 10^6^ cells/well in 6-well plates and incubated for 24 h at 37°C and 5% CO_2_. Various concentrations of the 3',4'-dihydroxyphenethyl anisate (**1**) (40 μM or 10 μM) were added. Following a 1-h pre-treatment, RAW 264.7 cells were stimulated with 6.25 ng/mL LPS for 24 h. Cells were washed with ice-cold DPBS and lysed in 250 μL of lysis buffer (50 mM Tris-HCl [pH 7.6], 150 mM NaCl, 1 mM phenylmethylsulfonyl fluoride [PMSF], 1% Nonidet P-40, 0.5% sodium deoxycholate, 0.1% sodium dodecyl sulfate [SDS], 0.5 mM ethylenediaminetetraacetic acid [EDTA], 2% β-mercaptoethanol, 10 μg/mL pepstatin A, and 10 μg/mL aprotinin). Lysates were incubated at 4°C for 15 min, then centrifuged at 13,000 × *g* for 10 min at 4°C. Supernatants were collected, and protein concentrations were determined using an enhanced BCA protein assay kit (Cyrusbioscience, Taiwan). 20 μg of proteins per sample were resolved by 8% sodium dodecyl sulfate-polyacrylamide gel electrophoresis (SDS-PAGE) and subsequently transferred to polyvinylidene fluoride (PVDF) membranes, which were pre-soaked in methanol for 1 minute, using a Trans-Blot^®^ SD semi-dry transblot module (Bio-Rad). Non-specific protein binding was blocked by incubating membranes with 5% bovine serum albumin (BSA) for 1 h at room temperature. Membranes were then incubated overnight at 4°C with primary antibodies against iNOS (ab3523, 1:1500) and GAPDH (ab8245, 1:2000) (Abcam, UK). After three washes with Tris-buffered saline containing 0.1% Tween 20 (TBST), the membranes were incubated for 1 h at room temperature with HRP-conjugated goat anti-rabbit IgG antibody (ab97051, 1:15000 dilution) (Abcam). Immunoreactive bands were detected using Western Lightning™ Pro Chemiluminescent Substrate (Revvity, USA) according to the manufacturer's instructions. Band densities were quantified using a Bio-Rad ChemiDoc™ XRS luminescent image analyzer and Image Lab version 6.0.1 software (Bio-Rad).

### Statistical Analysis

All data are presented as means ± standard deviations (SDs) from experiments conducted in triplicate. A statistical analysis was performed using Student’s *t*-test. Control reactions with LPS were used only for the anti-inflammatory assay, and reactions without the tested compounds were used for the anti-melanoma assay. A *p*-value of <0.05 was deemed statistically significant.

## Results

### Using the PDMA to Seek Novel Biotransformable Derivatives

**Fig. 1:**
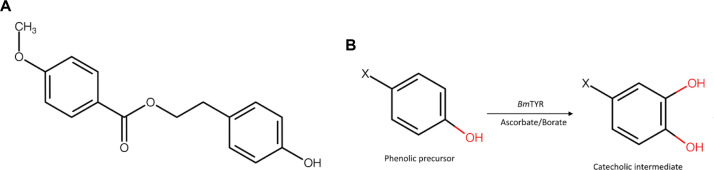
Design of biotransformation strategies and screening of the precursor compound using the PDMA. (A) Chemical structure of precursor, HP. (B) Enzymatic cascade biotransformation using *Bm*TYR.

**Fig. 2:**
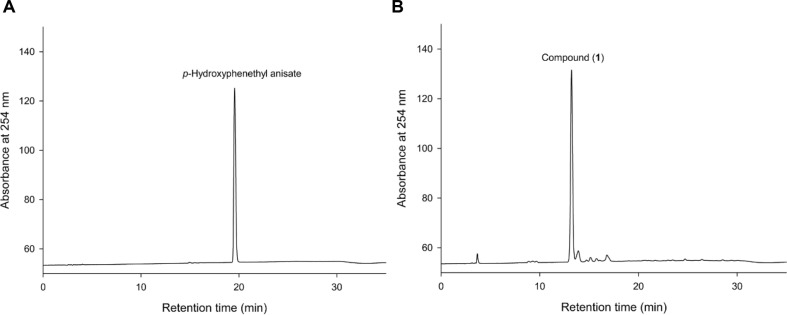
HPLC analysis of the biotransformation products of HP using *Bm*TYR (A, B). The biotransformation is described in the Materials and Methods section of the paper. At the end of the reaction, the reaction mixture was analyzed using HPLC, as described in the same section of the paper. (**A**) Precursor, p-hydroxyphenethyl anisate (HP). (**B**) Product, 3',4'-dihydroxyphenethyl anisate (**1**).

Based on the known capability of *Bm*TYR to catalyze the *ortho*-hydroxylation of phenolic compounds [13-16, 34, 35], we employed our previously reported PDMA [11] to screen for novel substrates. This strategy indicated that compounds mimicking the structure of tyrosine (Fig. 1B) could be converted to catechol products. The screening identified HP, a major constituent of the medicinal herb *Notopterygii rhizoma et Radix*, as a promising precursor (Fig. 1A). We then validated the predicted biotransformation of HP by *Bm*TYR to synthesize its novel hydroxylated derivative (Fig. 2).

### Novel Biotransformable Derivative from the Enzymatic Synthesis of p-Hydroxyphenethyl Anisate

**Fig. 3:**
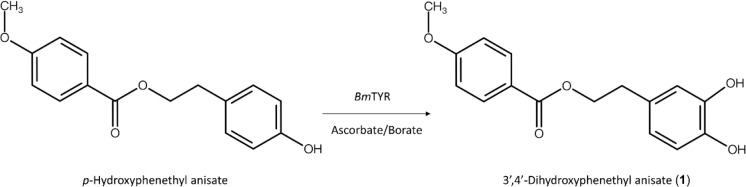
Biotransformation of HP using the *Bm*TYR reaction.

The chemical structure of purified compound (**1**) was then analyzed using high-resolution mass spectrometry (HRMS) and nuclear magnetic resonance (NMR) spectral analyses. The molecular formula of **1** was established as C_16_H_16_O_5_ by the HRMS at *m/z* 289.1067 [M+H]^+^ (Fig. S1). The ^1^H-NMR spectra (500 MHz, DMSO-*d6*) provided the following chemical shifts δ_H_: 2.82 (2H, t, *J* = 6.8 Hz, H-7'), 3.83 (3H, s, OCH_3_), 4.33 (2H, t, *J* = 6.8 Hz, H-8'), 6.52 (H, dd, *J* = 2.0, 8.0 Hz, H-6'), 6.65 (1H, d, *J* = 8.0 Hz, H-5'), 6.68 (1H, d, *J* = 2.0 Hz, H-2'), 7.03 (2H, dd, *J* = 2.7, 11.6 Hz, H-3, 5), and 7.88 (2H, dd, *J* = 2.7, 11.6 Hz, H-2, 6). The results observed in the ^13^C-NMR spectra (125 MHz, DMSO-*d6*) were as follows: δ_C_ 33.9 (C-7'), 55.5 (OCH_3_), 65.2 (C-8'), 114.0 (C-3, 5), 115.5 (C-5'), 116.2 (C-2'), 119.5 (C-6'), 122.0 (C-1), 128.7 (C-1'), 131.1 (C-2, 6), 143.7 (C-4'), 145.1 (C-3'), 163.1 (C-4), and 165.3 (C-7). The ^1^H-NMR spectra exhibited characteristic **1** a methoxy group [(δ_H_ 3.83, (3H, s, 4-OCH_3_)], and two methylene groups [(δ_H_ 2.82 (2H, t, *J* = 6.8 Hz, H-7'), 4.33 (2H, t, *J* = 6.8 Hz, H-8')], respectively. In the aromatic seven protons spectrum, four signal proton appeared as an AABB pattern at 7.03 (2H, dd, *J* = 2.7, 11.6 Hz) and 7.88 (2H, dd, *J* = 2.7, 11.6 Hz), assigned to H-2, H-3, H-5, and H-6, respectively, with the remaining three of the aromatic protons appeared as an A'B'X' pattern at 6.65 (d, *J* = 8.0 Hz), 6.52 (dd, *J* = 2.0, 8.0 Hz) and 6.68 (d, *J* = 2.0 Hz) assigned to H-5', H-6' and H-2', respectively. The structure of **1** was further demonstrated by analyses of 2D NMR spectra. The HMQC of **1** showed two methylene groups that there are correlation from δ_H_ 2.82 (H-7') and 4.33 (H-8') to carbonyl carbons δ_C_ 33.9 (C-7') and 65.2 (C-8'), respectively. The HMBC presented two methylene groups correlation from H-7' to C-1, C-2', C-6', and C-8' and H-8' to C-1', C-7' and C-7. In addition, the one aromatic ring of **1** substituent group a methoxy group at δ_H_ 3.83 (4-OCH_3_) was located at C-4 by the HMBC correlation from 4-OCH_3_ to C-4, another aromatic ring the substituents at C-3' and C-4' were identified as hydroxyl group because of its ^13^C-NMR chemical shift, that was located from C-3' to H-5' and from C-4' to H-2', H-6' by HMBC correlations. The complete assignments of the ^1^H and ^13^C- NMR signals were further aided by DEPT, HSQC, HMBC, COSY, and NOESY spectra, as shown in Figs. S2–S8. These data confirmed the structure of compound (**1**) to be 3',4'-dihydroxyphenethyl anisate (Fig. 3).

### Pharmacokinetic Profiles of 3',4'-Dihydroxyphenethyl Anisate for Bioavailability

Information on pharmacokinetic and toxicological properties is crucial for understanding the biological efficacy and safety of potential anti-inflammatory agents. Developing effective candidates necessitates that significant attention be given to their pharmacokinetic profiles, encompassing aspects such as intestinal absorption, overall safety, and skin permeability. In the present study, in silico toxicity predictions for 3',4'-dihydroxyphenethyl anisate (**1**) using pkCSM revealed no evidence of skin sensitization, minnow toxicity, or hepatotoxicity (Table 1). However, potential mutagenicity was predicted using pkCSM. We further investigated this by conducting, a comparative analysis with its precursor, HP, using VenomPred 2.0 (Table 2, Figs. S9 and S10). This comparison corroborated the pkCSM mutagenicity finding, as the predicted mutagenicity probability for 3',4'-dihydroxyphenethyl anisate (**1**) (28%) was twofold higher than that of HP (14%). Regarding hepatotoxicity, VenomPred 2.0 predicted a moderate probability for compound (**1**) (50%), slightly elevated from HP (46%). Notably, this high-probability prediction contradicts the negative hepatotoxicity result from the pkCSM model, indicating potential limitations of chemical structure-based prediction models. Collectively, these in silico findings suggest that while 3',4'-dihydroxyphenethyl anisate (**1**) exhibits favorable pharmacokinetic characteristics regarding water solubility and skin permeability, its extremely high predicted intestinal absorption and the potential mutagenicity risk—which was identified in two independent models—are key liabilities that must be prioritized in subsequent *in vitro* and *in vivo* safety evaluations.

### Preliminary Evaluation of the Anti-Melanoma Activity of 3',4'-Dihydroxyphenethyl Anisate

**Fig. 4:**
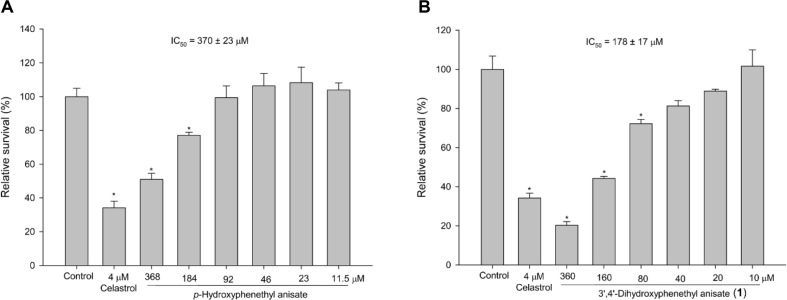
Anti-melanoma activities of (A) HP and (B) 3',4'-dihydroxyphenethyl anisate. B16 cells were treated with control vehicle (0.1% DMSO), celastrol (4 μM), indicated concentrations of HP or 3',4'-dihydroxyphenethyl anisate for 48 h. The amounts of viable cells were measured by MTT assay. The percentage of relative survival was calculated by setting vehicle control-treated group as 100% then mean ± SD plotted (*n* = 3). *p < 0.05, statistically significantly different from the value for cells treated in the control group.

The study utilized the PDMA strategy combined with enzymatic biotransformation to screen for bioactive derivatives from natural compounds. We initially assessed the anti-melanoma activity of HP and its biotransformed derivative, 3',4'-dihydroxyphenethyl anisate (**1**), using the murine B16 melanoma cell line. Consistent with previous reports, HP (a constituent of *Notopterygii rhizoma et Radix*) showed no significant cytotoxicity against the melanoma cells at the concentration till 184 µM (Fig. 4A). In contrast, observable inhibitory effects were detected for its *ortho*-hydroxylated product, compound (**1**), which exhibited an IC_50_ value of 178 ± 17 µM (Fig. 4B). These results indicate that the introduction of an *ortho*-hydroxyl group represents a fundamental structural feature contributing to the observed anti-melanoma activity.

### 3',4'-Dihydroxyphenethyl Anisate Possesses Potent Antioxidant Activity

**Fig. 5:**
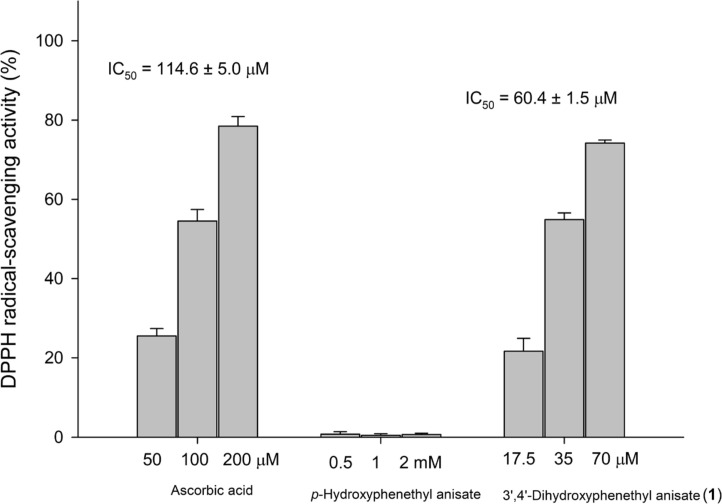
DPPH free radical-scavenging activity of ascorbic acid, HP, and its derivative, 3',4'-dihydroxyphenethyl anisate. DPPH scavenging activity was determined as described in the Materials and Methods section of the paper. The mean (*n* = 3) is shown, and SDs are represented by error bars. The IC_50_ values represent the concentrations required to scavenge DPPH free radical by 50%.

It is well established that the *ortho*-dihydroxyl (catechol) groups on the benzene ring of phenolic structures are crucial for mediating antioxidant activity [10, 21, 36]. Therefore, the antioxidant capacities of 3',4'-dihydroxyphenethyl anisate (**1**) and its precursor, HP, were evaluated using the DPPH free radical scavenging assay. The compounds were tested across a range of concentrations. The results revealed that the precursor, HP, displayed no significant radical scavenging activity at the tested concentrations. In stark contrast, the *ortho*-hydroxylated product, 3',4'-dihydroxyphenethyl anisate, demonstrated clear dose-dependent antioxidant activity, with a calculated IC_50_ value of 60.4 ± 1.5 µM (Fig. 5). These findings confirm that enzymatic hydroxylation successfully conferred novel antioxidant properties to the parent molecule.

### 3',4'-Dihydroxyphenethyl Anisate Possess Potent Anti-NO Activity

**Fig. 6:**
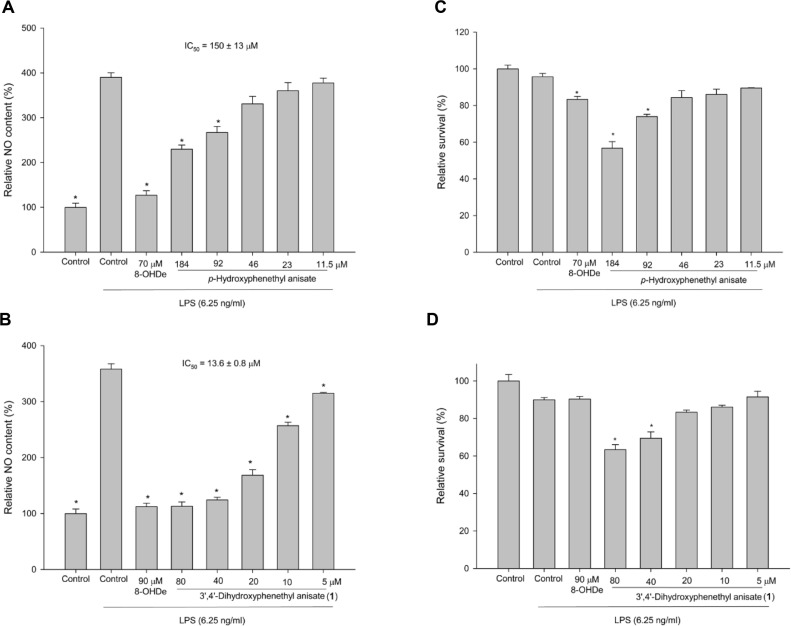
Effects of HP (A, C) and 3',4'-dihydroxyphenethyl anisate (B) on the inhibition of LPS-induced NO production (B) and cell survival (C, D) in murine macrophage RAW 264.7 cells. The cells were incubated with compounds at the indicated concentrations for 1 h before treatment with LPS (6.25 ng/mL) for 24 h. The amounts of NO were measured using Griess reagent in the culture medium. Cell viability was determined using MTT assay. Each value indicates the mean ± SD and is representative of the results obtained from three independent experiments. *p < 0.05, statistically significantly different from the value for the cells treated with LPS.

Based on reports that catechol structures are critical for anti-inflammatory activity [24, 37-39], we hypothesized that the biotransformation of HP to 3',4'-dihydroxyphenethyl anisate (**1**) would enhance its anti-inflammatory potential. To test this, the compounds were assayed for anti-NO activity. The *ortho*-hydroxylated product (**1**) exhibited an IC_50_ of 13.6 ± 0.8 μM for NO production inhibition, representing an 11-fold improvement over its precursor (HP) (Fig. 6A and 6B). These concentrations of 3',4'-dihydroxyphenethyl anisate showed significant inhibitory activity with minor cytotoxicity on RAW 264.7 cells (Fig. 6D). This confirms that enzymatic *ortho*-hydroxylation successfully enhanced the compound's anti-inflammatory properties.

### 3',4'-Dihydroxyphenethyl Anisate Inhibits iNOS, COX-2, and TNF-α Expression in LPS-Stimulated RAW 264.7 Cells

**Fig. 7:**
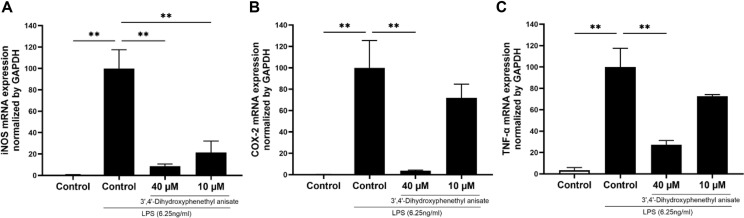
Effects of 3',4'-dihydroxyphenethyl anisate on LPS-induced mRNA expression of iNOS (A), COX-2 (B) and TNF-α (C) in RAW 264.7 cells. The cells were not treated or were pretreated with 3',4'-dihydroxyphenethyl anisate (40 or 10 μM) for 1 h prior to being stimulated with LPS (6.25 ng/mL) for 24 h. Total RNA was prepared from cells pretreated with or without the indicated concentrations of 3',4'-dihydroxyphenethyl anisate for 1 h and then stimulated with LPS (6.25 ng/mL) for 24 h. The mRNA levels of iNOS, COX-2 and TNF-α were determined using qPCR, and the values were normalized to GAPDH. Each value indicates the mean ± SD and is representative of the results obtained from three independent experiments. *p < 0.05 and **p < 0.01 statistically significantly different from the value for the cells treated with LPS.

Given the significantly enhanced anti-NO activity of 3',4'-dihydroxyphenethyl anisate (**1**) over its precursor HP, we investigated its underlying anti-inflammatory mechanism at the transcriptional level. The upregulation of key pro-inflammatory mediators, including inducible iNOS, COX-2, and TNF-α, are hallmarks of the inflammatory cascade[40-43]. We found that 3',4'-dihydroxyphenethyl anisate (**1**) effectively suppressed the gene expression of these mediators in LPS-stimulated RAW 264.7 cells. Treatment with 3',4'-dihydroxyphenethyl anisate significantly and dose-dependently decreased the mRNA levels of iNOS (Fig. 7A), COX-2 (Fig. 7B), and TNF-α (Fig. 7C). These findings collectively demonstrate that 3', 4'-dihydroxyphenethyl anisate mitigates the LPS-induced inflammatory response by suppressing key pro-inflammatory enzymes and cytokines at the mRNA level.

**Fig. 8:**
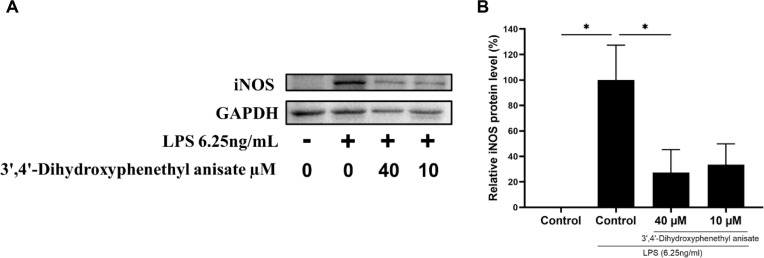
Effects of 3',4'-dihydroxyphenethyl anisate on LPS-induced protein expression of iNOS. The cells were incubated with 3',4'-dihydroxyphenethyl anisate at the indicated concentrations for 1 h before treatment with LPS (6.25 ng/mL) for 24 h. (**A**) Representative Western blot showing iNOS and GAPDH (loading control) protein levels. (**B**) Relative quantification of iNOS protein expression normalized to GAPDH. Each value indicates the mean ± standard deviation (SD). *p < 0.05 statistically significantly different from the control group treated with LPS.

To further validate the anti-inflammatory mechanism at the protein level, Western blot analysis was performed to evaluate the expression of iNOS. As shown in Fig. 8A, LPS treatment significantly induced iNOS protein expression in RAW 264.7 cells. However, treatment with 3',4'-dihydroxyphenethyl anisate effectively suppressed this induction. Quantitative analysis revealed that 3',4'-dihydroxyphenethyl anisate at 40 μM and 10 μM reduced iNOS protein levels by approximately 72 % and 66 %, respectively, compared to the LPS-stimulated RAW 264.7 cells (Fig. 8B). These results provide robust evidence that 3',4'-dihydroxyphenethyl anisate exerts potent anti-inflammatory activity by modulating key inflammatory mediators.

### 3',4'-Dihydroxyphenethyl Anisate Inhibits the Production of IL-6 and IL-1β in LPS-Stimulated RAW 264.7 Cells

**Fig. 9:**
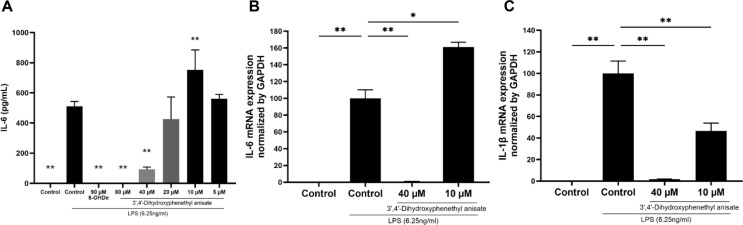
Effects of 3',4'-dihydroxyphenethyl anisate on the expression of IL-1β and IL-6. The cells were incubated with 3',4'-dihydroxyphenethyl anisate at the indicated concentrations for 1 h before treatment with LPS (6.25 ng/mL) for 24 h. (**A**) The production of IL-6 in the culture medium was assayed by ELISA. (**B–C**) Relative mRNA expression of IL-6 and IL-1β. Each value indicates the mean ± SD and is representative of the results obtained from three independent experiments. *p < 0.05 and **p < 0.01 statistically significantly different from the value for the cells treated with LPS.

The anti-inflammatory effects of 3',4'-dihydroxyphenethyl anisate were further evaluated by examining its impact on the pro-inflammatory cytokines IL-6 and IL-1β, both critical mediators of inflammation [41, 44]. We assessed IL-6 protein secretion via ELISA and the mRNA expression of both cytokines via qPCR. Treatment with 3',4'-dihydroxyphenethyl anisate caused a significant and dose-dependent reduction in IL-6 protein secretion compared to the LPS-stimulated control (Fig. 9A). This finding was consistent at the transcriptional level, where 3',4'-dihydroxyphenethyl anisate also downregulated the mRNA expression of IL-6 (Fig. 9B) and IL-1β (Fig. 9C). These data demonstrate that 3',4'-dihydroxyphenethyl anisate suppresses both the expression and secretion of IL-6 and IL-1β, thereby impeding inflammation.

## Discussion

The present study employed the PDMA to identify novel compounds originating from HP. We initiated this work by establishing criteria for a target biotransformation, and subsequently utilized the PDMA to screen for precursors that could be converted into novel chemical entities. This process successfully identified HP as a candidate, from which a new derivative was generated via enzymatic biotransformation. The bioactivities of this purified product were then characterized and confirmed through *in vitro* assays.

In this study, we demonstrated the successful biotransformation of HP by *Bm*TYR into its novel catechol product, 3',4'-dihydroxyphenethyl anisate (Fig. 3). The identification of this product as a novel compound validates the efficacy of the PDMA. This supports the value of the approach in predicting substrates for various enzymatic reactions, including hydroxylations [17, 18, 45, 46] and glycosylations [33, 47].The introduction of the *ortho*-dihydroxyl (catechol) group was hypothesized to enhance bioactivity, as this moiety is critical for antioxidant [10, 21, 36] and anti-inflammatory functions [24, 37-39]. Our results partially support this hypothesis; the novel product (**1**) exhibited significant anti-inflammatory activity (Figs. 6–9), while preliminary antioxidant and anti-melanoma effects were also observed (Figs. 4 and 5). Our findings suggest that the *ortho*-hydroxylation of HP serves as a key structural modification that enhances its biological potential compared to the precursor. Although the specific mechanisms underlying the observed anti-melanoma activity and the potential interplay between the antioxidant, anti-inflammatory, and anti-melanoma effects remain to be fully elucidated, the successful identification of this previously unreported metabolite underscores the efficiency of the PDMA strategy. Future work will focus on optimizing this process by incorporating in silico molecular docking simulations [48, 49] to better predict biotransformation efficiency and bioactivity, thereby reducing experimental costs and resource consumption.

Our data provide further insights into the potential mechanism behind the enhanced anti-inflammatory activity of 3',4'-dihydroxyphenethyl anisate. We experimentally confirmed that 3',4'-dihydroxyphenethyl anisate significantly inhibits NO production (Fig. 6). A plausible mechanism, consistent with the activity of many phenolic compounds, is that 3',4'-dihydroxyphenethyl anisate probably exhibits anti-inflammatory activity by inhibiting the phosphorylation of mitogen-activated protein kinases (MAPKs) and the activation of NF-κB in LPS-activated RAW 264.7 cells [50]. This upstream modulation suppresses the expression of key inflammatory enzymes [50]. Our results support this, as we observed a significant, dose-dependent downregulation of both iNOS and COX-2 mRNA (Fig. 7A and 7B). Correspondingly, Western blot analysis further confirmed that this inhibitory effect extends to the protein level, where 3',4'-dihydroxyphenethyl anisate significantly suppressed iNOS protein expression (Fig. 8). This suppression subsequently leads to a reduction in NO and prostaglandin E_2_ (PGE_2_) production [42, 43, 51], aligning with our confirmed data on NO inhibition. This suppressive action also extends to key pro-inflammatory cytokines. 3',4'-dihydroxyphenethyl anisate significantly reduced the secretion of IL-6 protein (ELISA, Fig. 9A). This finding was consistent at the transcriptional level, where 3',4'-dihydroxyphenethyl anisate also suppressed the mRNA expression of TNF-α (Fig. 7C), IL-1β (Fig. 9C), and IL-6 (Fig. 9B). These mediators are crucial in inflammatory signaling [41, 44]. Collectively, the evidence suggests that 3',4'-dihydroxyphenethyl anisate effectively mitigates the inflammatory response. Therefore, 3',4'-dihydroxyphenethyl anisate demonstrates considerable anti-inflammatory potential, and its precise molecular targets in the MAPK and NF-κB pathways warrant further investigation.

A critical point of discussion is the structure–activity relationship of 3',4'-dihydroxyphenethyl anisate. We hypothesize that its broad bioactivity spectrum is linked to its structural similarity to hydroxytyrosol (HT). HT is a well-established natural compound, and its potent anti-inflammatory [52-54], antioxidant [52-55], and anti-melanoma [52, 56] activities have been widely documented. Structurally, 3',4'-dihydroxyphenethyl anisate may be considered an ester derivative of HT. The established mechanisms for HT often involve the inhibition of the MAPK and NF-κB signaling pathways, which subsequently suppresses iNOS, COX-2, and pro-inflammatory cytokines [52-54]. This established profile plausibly explains the anti-inflammatory effects we observed for 3',4'-dihydroxyphenethyl anisate. Specifically, the compound potently inhibited NO secretion and comprehensively downregulated iNOS, COX-2, TNF-α, IL-6, and IL-1β mRNA. Therefore, the 3',4'-dihydroxyphenethyl structure, common to both molecules, likely underlies the observed activities. This suggests that 3',4'-dihydroxyphenethyl anisate is a promising candidate whose precise anti-inflammatory mechanism warrants further investigation.

## Conclusion

We have successfully generated a novel catechol derivative via enzymatic biotransformation of a known precursor identified using our PDMA. This study demonstrates that targeted *ortho*-hydroxylation can be employed to confer potent new biological functions. The novel product has marked anti-inflammatory effects and preliminary potential as an antioxidant and anti-melanoma agent. Mechanistic insights suggest that the compound acts by suppressing multiple pro-inflammatory mediators at the transcriptional level. This profile is likely related to its core chemical structure, which is similar to other known potent phenols, such as hydroxytyrosol. This research validates the PDMA for screening both natural and chemically synthesized compounds and presents a promising new candidate for pharmacological development. Future studies should explore its mechanisms in greater detail and evaluate the potential to scale this biotransformation for industrial applications.

## Supplemental Materials

Supplementary data for this paper are available on-line only at http://jmb.or.kr.



## Figures and Tables

**Table 1:** In silico pharmacokinetic and toxicity profiles of 3',4'-dihydroxyphenethyl anisate generated via biotransformation determined using pkCSM.

Property	Predicted Value	Desired Value
Aqueous solubility (log mol/L)	-3.206	> -4 (moderate solubility)
Human intestinal absorption (%)	95.361	> 70% (well absorbed)
Skin permeability (Log Kp)	-2.988	< -2.5 (low permeability)
Minnow toxicity (log mM)	-0.248	> -0.3 (low acute toxicity)
Skin sensitization	No	No
Mutagenicity	Yes	Yes
Hepatotoxicity	No	No

**Table 2:** Comparative in silico toxicological predictions of HP (*p*-hydroxyphenethyl anisate) and its biotransformed derivative, 1 (3',4'-dihydroxyphenethyl anisate), via VenomPred 2.0.

Endpoint	Predicted Probability	
	HP	1
Mutagenicity	14%	28%
Carcinogenicity	41%	28%
Hepatotoxicity	46%	50%
Estrogenicity	51%	54%
Androgenicity	3%	3%
Acute oral toxicity	10%	12%
Skin irritation	10%	9%
Eye irritation	11%	12%

## References

[1] Choudhary M, Gupta S, Dhar MK, Kaul S (2021). Endophytic fungi-mediated biocatalysis and biotransformations paving the way toward green chemistry. Front. Bioeng. Biotechnol..

[2] Klein C, Hüttel W (2011). A simple procedure for selective hydroxylation of L-proline and L-pipecolic acid with recombinantly expressed proline hydroxylases. Adv. Synth. Catal..

[3] Shanu-Wilson J, Evans L, Wrigley S, Steele J, Atherton J, Boer J (2020). Biotransformation: impact and application of metabolism in drug discovery. ACS Med. Chem. Lett..

[4] Li C, Yang K, Li H, Jia M, Guan L, Qin H-M (2023). Editorial: biocatalysis and biotransformation guided by protein engineering. Front. Bioeng. Biotechnol..

[5] Fryszkowska A, Devine PN (2020). Biocatalysis in drug discovery and development. Curr. Opin. Chem. Biol..

[6] Giri A, Dhingra V, Giri CC, Singh A, Ward OP, Narasu ML (2001). Biotransformations using plant cells, organ cultures and enzyme systems: current trends and future prospects. Biotechnol. Adv..

[7] Banerjee S, Singh S, Rahman LU (2012). Biotransformation studies using hairy root cultures - A review. Biotechnol. Adv..

[8] Cao H, Chen X, Jassbi AR, Xiao J (2015). Microbial biotransformation of bioactive flavonoids. Biotechnol. Adv..

[9] de Matos IL, Nitschke M, Porto ALM (2023). Regioselective and chemoselective biotransformation of 2'-hydroxychalcone derivatives by marine-derived fungi. Biocatal. Biotransform..

[10] Zafar S, Ahmed R, Khan R (2016). Biotransformation: a green and efficient way of antioxidant synthesis. Free Radic. Res..

[11] Wu J-Y, Ding H-Y, Wang T-Y, Cai C-Z, Chang T-S (2023). Antioxidant and anti-α-glucosidase activities of biotransformable dragon's blood via predicted data mining approach. Process Biochem..

[12] Chang T-S, Ding H-Y, Wu J-Y, Wang M-L, Ting H-J (2023). Biotransformation-guided purification of a novel glycoside derived from the extracts of Chinese herb Baizhi. J. Biosci. Bioeng..

[13] Chiang C-M, Wang D-S, Chang T-S (2016). Improving free radical scavenging activity of soy isoflavone glycosides daidzin and genistin by 3'-hydroxylation using recombinant Escherichia coli. Molecules.

[14] Wu J-Y, Ding H-Y, Wang T-Y, Cai C-Z, Chang T-S (2022). Application of biotransformation-guided purification in chinese medicine: an example to produce butin from licorice. Catalysts.

[15] Wu J-Y, Ding H-Y, Wang T-Y, Hsu M-H, Chang T-S (2022). A new stilbene glucoside from biotransformation-guided purification of Chinese herb Ha-Soo-Oh. Plants.

[16] Wu J-Y, Wang T-Y, Ding H-Y, Lee C-C, Chang T-S (2022). A novel soy isoflavone derivative, 3'-hydroyxglcitin, with potent antioxidant and anti-α-glucosidase activity. Plants.

[17] Wu C-Y, Ding H-Y, Wang T-Y, Liu C-W, Wu J-Y, Chang T-S (2024). Development of a new isoxsuprine hydrochloride-based hydroxylated compound with potent antioxidant and anti-inflammatory activities. J. Microbiol. Biotechnol..

[18] Wu C-Y, Wu J-Y, Muddasir K, Ding H-Y, Wang T-Y, Huang H-L (2025). Synthesis of a novel bioactive compound by biotransformation of 4-[(2',4'-dinitrophenyl)amino]-phenol (DPAP) via predicted data mining approach (PDMA). Biocatal. Biotransform..

[19] Holland HL, Weber HK (2000). Enzymatic hydroxylation reactions. Curr. Opin. Biotechnol..

[20] Liu Y, Qian J, Li J, Xing M, Grierson D, Sun C (2022). Hydroxylation decoration patterns of flavonoids in horticultural crops: chemistry, bioactivity, and biosynthesis. Hortic. Res.-England..

[21] Sekher Pannala A, Chan TS, O'Brien PJ, Rice-Evans CA (2001). Flavonoid B-ring chemistry and antioxidant activity: fast reaction kinetics. Res. Commun..

[22] Xiao J, Kai G, Yamamoto K, Chen X (2013). Advance in dietary polyphenols as α-glucosidases inhibitors: a review on structure-activity relationship aspect. Crit. Rev. Food Sci. Nutr..

[23] Xiao J, Ni X, Kai G, Chen X (2013). A review on structure-activity relationship of dietary polyphenols inhibiting α-amylase. Crit. Rev. Food Sci. Nutr..

[24] Yan S, Lin H, Huang H, Yang M, Xu B, Chen G (2019). Microbial hydroxylation and glycosidation of oleanolic acid by *Circinella muscae* and their anti-inflammatory activities. Nat. Prod. Res..

[25] Kozawa M, Fukumoto M, Matsuyama Y, Baba K (1983). Chemical studies on the constituents of the chinese crude drug "Quiang Huo". Chem. Pharm. Bull..

[26] Li Y-H, Luo F, Peng S-L, Liang J, Ding L-S (2006). A new dihydroisocoumarin from the rhizomes of *Notopterygium forbesii*. Nat. Prod. Res..

[27] Azietaku JT, Ma H, Yu X-a, Li J, Oppong MB, Cao J (2017). A review of the ethnopharmacology, phytochemistry and pharmacology of *Notopterygium incisum*. J. Ethnopharmacol..

[28] Liu Z-W, Zhou J (2024). DNA barcoding of *Notopterygii Rhizoma et Radix* (Qiang-huo) and identification of adulteration in its medicinal services. Sci. Rep..

[29] Chang T-S, Wu J-Y, Ding H-Y, Wang T-Y, Chen J-Y, Ting H-J (2025). Chromatography-guided purification and characterization of *p*-hydroxyphenethyl anisate as a potent anti-melanogenesis component from the concentrated Chinese medicine, Qiang Huo. Prep. Biochem. Biotechnol..

[30] Chang T-S, Wang T-Y, Yang S-Y, Kao Y-H, Wu J-Y, Chiang C-M (2019). Potential industrial production of a well-soluble, alkaline-stable, and anti-inflammatory isoflavone glucoside from 8-hydroxydaidzein glucosylated by recombinant amylosucrase of *Deinococcus geothermalis*. Molecules.

[31] Pires DEV, Blundell TL, Ascher DB (2015). pkCSM: Predicting small-molecule pharmacokinetic and toxicity properties using graph-based signatures. J. Med. Chem..

[32] Di Stefano M, Galati S, Piazza L, Granchi C, Mancini S, Fratini F (2024). VenomPred 2.0: a novel in silico platform for an extended and human interpretable toxicological profiling of small molecules. J. Chem Inf. Model..

[33] Chang T-S, Wu J-Y, Ding H-Y, Tayo LL, Suratos KS, Tsai P-W (2025). Predictive production of a new highly soluble glucoside, corylin-7-*o*-*β*-glucoside with potent anti-inflammatory and anti-melanoma activities. Appl. Biochem. Biotechnol..

[34] Lee S-H, Baek K, Lee J-E, Kim B-G (2016). Using tyrosinase as a monophenol monooxygenase: a combined strategy for effective inhibition of melanin formation. Biotechnol. Bioeng..

[35] Chang T-S (2009). An updated review of tyrosinase inhibitors. Int. J. Mol. Sci..

[36] Treml J, Šmejkal K (2016). Flavonoids as potent scavengers of hydroxyl radicals. Compr. Rev. Food. Sci. Food Saf..

[37] Luo D, Or TCT, Yang CLH, Lau ASY (2014). Anti-Inflammatory Activity of Iridoid and Catechol Derivatives from *Eucommia ulmoides* Oliver. ACS Chem. Neurosci..

[38] Shin HS, Satsu H, Bae M-J, Totsuka M, Shimizu M (2017). Catechol groups enable reactive oxygen species scavenging-mediated suppression of PKD-NFkappaB-IL-8 signaling pathway by chlorogenic and caffeic acids in human intestinal cells. Nutrients.

[39] Zheng LT, Ryu G-M, Kwon B-M, Lee W-H, Suk K (2008). Anti-inflammatory effects of catechols in lipopolysaccharide-stimulated microglia cells: Inhibition of microglial neurotoxicity. Eur. J. Pharmacol..

[40] Lee S-J, Rim H-K, Jung J-Y, An H-J, Shin J-S, Cho C-W (2013). Immunostimulatory activity of polysaccharides from Cheonggukjang. Food Chem. Toxicol..

[41] Li K, Hu W, Yang Y, Wen H, Li W, Wang B (2023). Anti-inflammation of hydrogenated isoflavones in LPS-stimulated RAW264.7 cells via inhibition of NF-κB and MAPK signaling pathways. Mol. Immunol..

[42] Pérez-Sala D, Lamas S (2001). Regulation of cyclooxygenase-2 expression by nitric oxide in cells. Antioxid. Redox Signal..

[43] Kim JM, Heo HJ (2022). The roles of catechins in regulation of systemic inflammation. Food Sci. Biotechnol..

[44] Wu Z, Wang D, Liu C-X, Wang X-H, Chen Y, Wu Q-X (2022). Macrophage immunity promotion effect of polysaccharide LGP-1 from Guapian tea via PI3K/AKT and NF-κB signaling pathway. J. Funct. Food.

[45] Chang T-S, Wu J-Y, Ding H-Y, Wang T-Y, Liu G-C, Wang M-L (2025). Enzymatic synthesis of a new and bioactive dihydrochalcone: 3,4-dihydroxy-2?,6?-dimethoxy dihydrochalcone. Nat. Prod. Res..

[46] Wu C-Y, Khan M, Ding H-Y, Wang T-Y, Li Y-X, Liu C-W (2025). Biotransformation: a novel approach for the production of 6-hydroxyluteolin 7-*O*-methylglucuronide compound with antioxidant and anti-inflammatory potential. Nat. Prod. Res..

[47] Chang T-S, Ding H-Y, Wang T-Y, Wu J-Y, Tsai P-W, Suratos KS (2025). In silico-guided synthesis of a new, highly soluble, and anti-melanoma flavone glucoside: Skullcapflavone II-6'-*O*-*β*-glucoside. Biotechnol. Appl. Biochem..

[48] Garcia PJB, Huang SK-H, De Castro-Cruz KA, Leron RB, Tsai P-W (2023). *In silico* neuroprotective effects of specific *Rheum palmatum* metabolites on Parkinson's disease targets. Int. J. Mol. Sci..

[49] Sobremisana G, Ferrer R, Carpio AR, Tayo LL, Tsai P-W, Hsueh C-C (2023). Exploring characteristics of value-added production of anthraquinones in rhubarb via fermentation: Compartmental modelling and molecular docking analysis. J. Taiwan Inst. Chem. Eng..

[50] Hung Y-L, Fang S-H, Wang S-C, Cheng W-C, Liu P-L, Su C-C (2017). Corylin protects LPS-induced sepsis and attenuates LPS-induced inflammatory response. Sci. Rep..

[51] Upadhyay A, Ayeman A, Vibhuti J, Rohan D, Kumar PV, Mohan PK (2021). Ibuprofen-based advanced therapeutics: breaking the inflammatory link in cancer, neurodegeneration, and diseases. Drug Metab. Rev..

[52] Karković Marković A, Torić J, Barbarić M, Jakobušić Brala C (2019). Hydroxytyrosol, tyrosol and derivatives and their potential effects on human health. Molecules.

[53] Batarfi WA, Yunus MHM, Hamid AA, Lee YT, Maarof M (2024). Hydroxytyrosol: a promising therapeutic agent for mitigating inflammation and apoptosis. Pharmaceutics.

[54] Fuccelli R, Fabiani R, Rosignoli P (2018). Hydroxytyrosol exerts anti-inflammatory and anti-oxidant activities in a mouse model of systemic inflammation. Molecules.

[55] D'Angelo S, Ingrosso D, Migliardi V, Sorrentino A, Donnarumma G, Baroni A (2005). Hydroxytyrosol, a natural antioxidant from olive oil, prevents protein damage induced by long-wave ultraviolet radiation in melanoma cells. Free Radical Biol. Med..

[56] Costantini F, Di Sano C, Barbieri G (2020). The hydroxytyrosol induces the death for apoptosis of human melanoma cells. Int. J. Mol. Sci..

