# Supplementary material for: Prediction-Guided Biotransformation of p-Hydroxyphenethyl Anisate into a Novel 3',4'-Dihydroxyphenethyl Anisate with Potent Anti-Inflammatory, Anti-Melanoma, and Antioxidant Activities
Source: J Microbiol Biotechnol. 2026 May 11;36:e2601040. doi: 10.4014/jmb.2601.01040 (PMC13173354; doi:10.4014/jmb.2601.01040)
Supplement: Supplementary file 1 [file jmb-36-e2601040-supple.pdf]

J. Microbiol. Biotechnol. 2026. 36: e2601040

https://doi.org/10.4014/jmb.2601.01040

Supplementary Figures

Prediction-guided biotransformation of *p*-hydroxyphenethyl anisate into a novel 3', 4'-dihydroxyphenethyl anisate with potent anti-inflammatory, anti-melanoma, and antioxidant activities

Chien-Yu Wu[a,b], Hsiou-Yu Ding[c], Huei-Ju Ting[a], Tzi-Yuan Wang[d], Tzu-Hsuan Li[a], Jou-Yi Chen[a], Hui-Ling Huang[a], Jiumn-Yih Wu[e*] and Te-Sheng Chang[a*]

[a]Department of Biological Sciences and Technology, National University of Tainan, Tainan 700301, Taiwan. [b]The integrative Evolutionary Galliform Genomics (iEGG) and Animal Biotechnology Center, National Chung Hsing University, Taichung 402202, Taiwan. [c]Department of Cosmetic Science, Chia Nan University of Pharmacy and Science, Tainan 717301, Taiwan. [d]Biodiversity Research Center, Academia Sinica, Taipei 115201, Taiwan. [e]Department of Food Science, National Quemoy University, Kinmen County 892009, Taiwan.

Chien-Yu Wu: gtm310050@gmail.com / Hsiou-Yu Ding: ding8896@gmail.com

Huei-Ju Ting: hting@mail.nutn.edu.tw / Tzi-Yuan Wang: tziyuan@gmail.com; sds@gate.sinica.edu.tw / Tzu-Hsuan Li: ql20001128@gmail.com

Jou-Yi Chen: chenruyi000@gmail.com / Hui-Ling Huang: Michel.liu02@gmail.com

*Corresponding author: Te-Sheng Chang, mozyme2001@gmail.com; cts@mail.nutn.edu.tw;

Abstract

Discovering novel bioactive compounds is a fundamental goal in scientific research. To this end, our study employed a predicted data mining approach (PDMA) to efficiently screen for biotransformable precursors capable of yielding new bioactive derivatives. Using the PDMA strategy, we identified *p*-hydroxyphenethyl anisate (HP) as a substrate and employed *Bacillus megaterium* tyrosinase (*Bm*TYR) to catalyze the hydroxylation of HP. The results demonstrated that HP was successfully converted into a novel ortho-dihydroxyphenyl compound, 3', 4'-dihydroxyphenethyl anisate. This biotransformation yielded a product with significantly enhanced therapeutic potential. Notably, the product showed a potent 11-fold increase in anti-inflammatory activity compared to the precursor. Mechanistically, 3', 4'-dihydroxyphenethyl anisate effectively mitigated the hyperimmune response in lipopolysaccharide-stimulated RAW 264.7 macrophages by suppressing the gene expression of pro-inflammatory mediators, including tumor necrosis factor-alpha, interleukin-1 beta, interleukin 6, inducible nitric oxide synthase, and cyclooxygenase-2. Furthermore, the hydroxylated product showed significant antioxidant capacity (absent in the parent compound) and markedly higher anti-melanoma activity. This study validates PDMA as an effective strategy for generating novel, high-value bioactive molecules via biotransformation. Our newly produced catechol is a promising candidate for future pharmacological applications.

Keywords: Anti-inflammation, Anti-melanoma, Antioxidant, Hydroxylation, Predicted data mining approach, Biotransformation

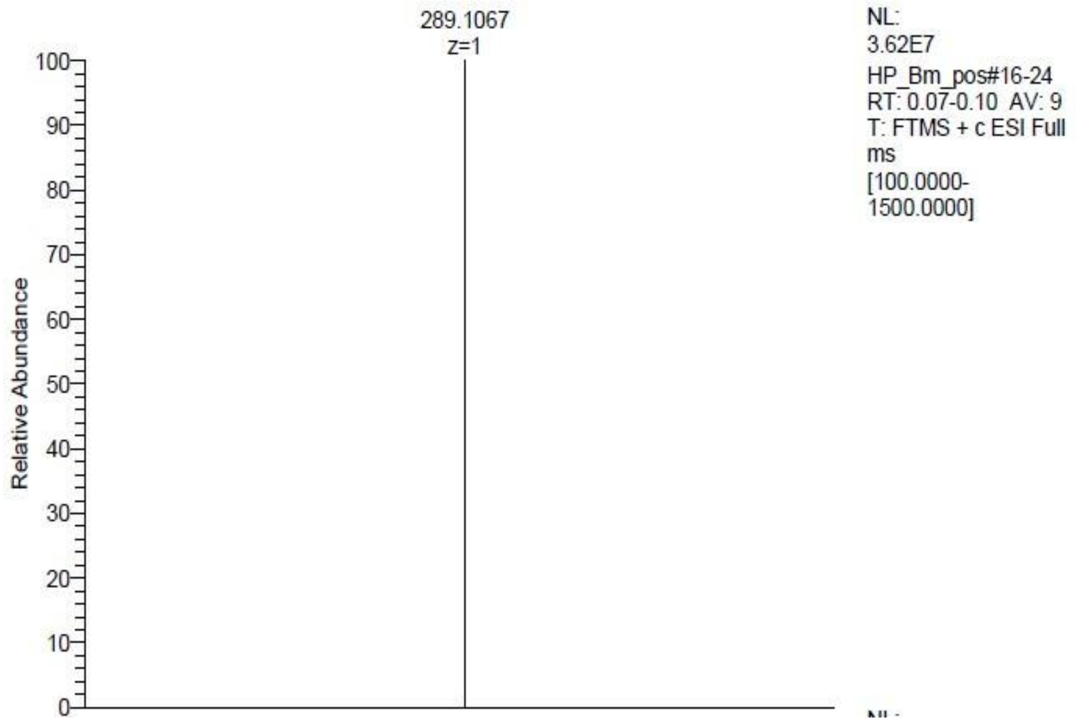

Fig. S1. The high-resolution mass spectrometry analysis of 3', 4'-dihydroxyphenethylanisate (1) at the positive mode. A significant signal at m/z 289.1067.

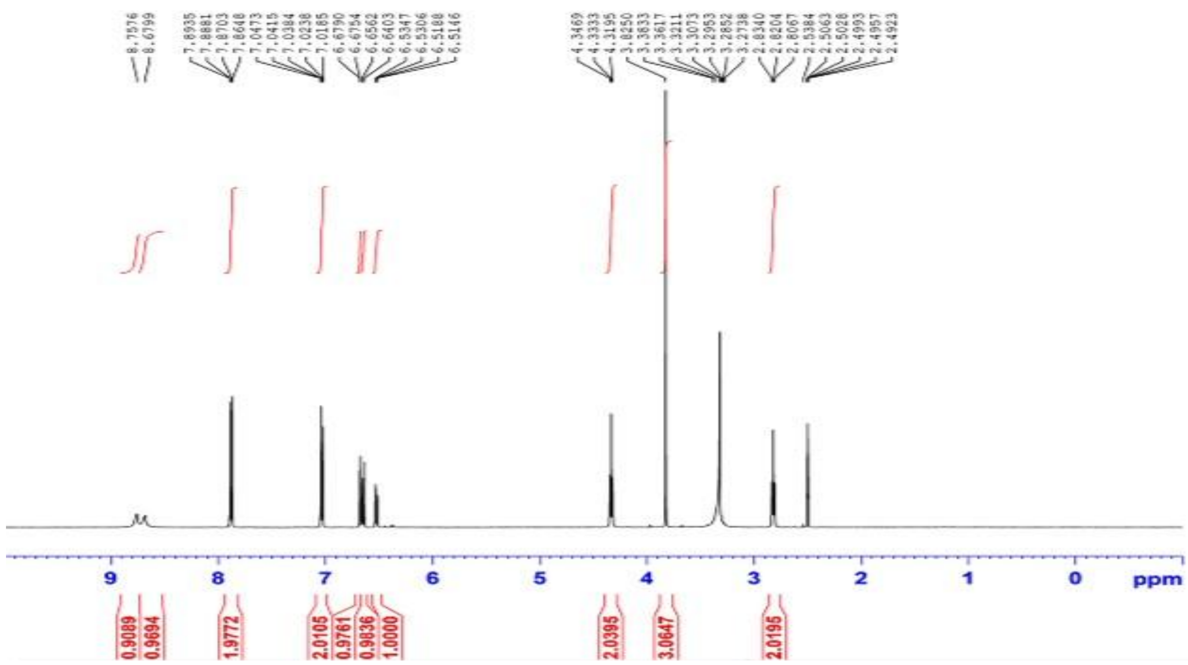

Fig. S2. 1D NMR spectrum (^{1}H-NMR, 500 MHz, DMSO-*d*$_6$) of the 3', 4'-dihydroxyphenethylanisate (1).

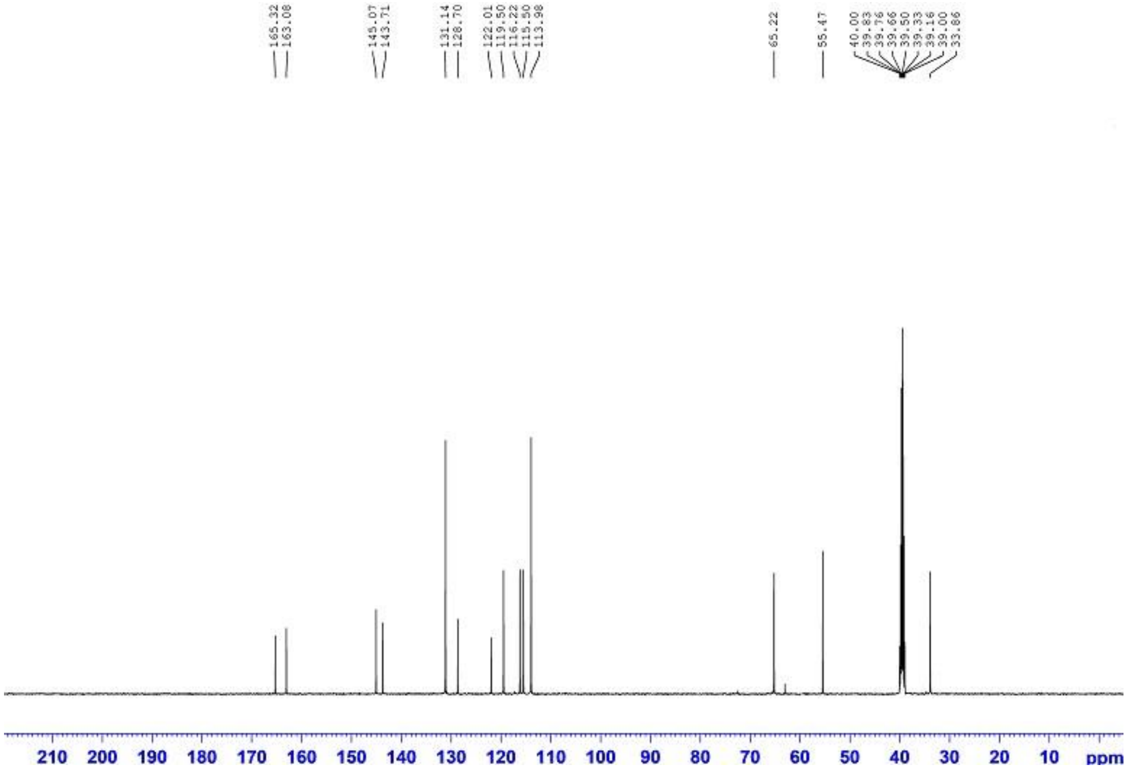

Fig. S3. 1D NMR spectrum (^{13}C-NMR, 125 MHz, DMSO-d_6) of the 3', 4'-dihydroxyphenethylanisate (1).

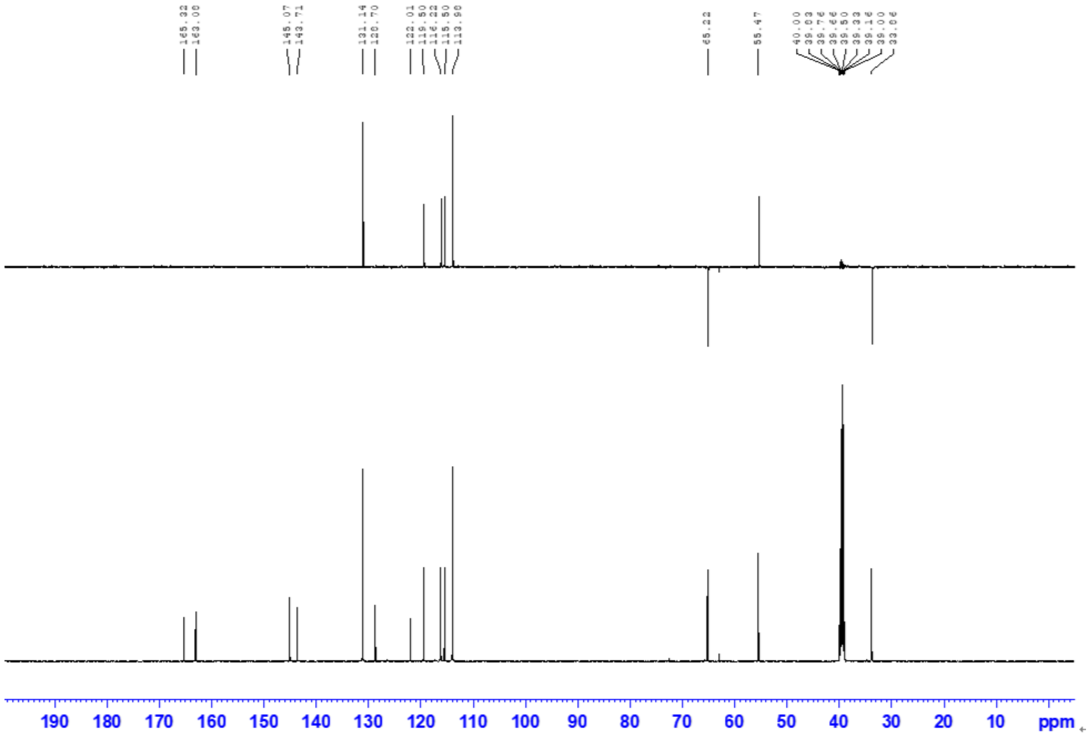

Fig. S4. 1D NMR spectrum (DEPT-135, 125 MHz, DMSO-*d₆*) of the 3', 4'-dihydroxyphenethylanisate (1).

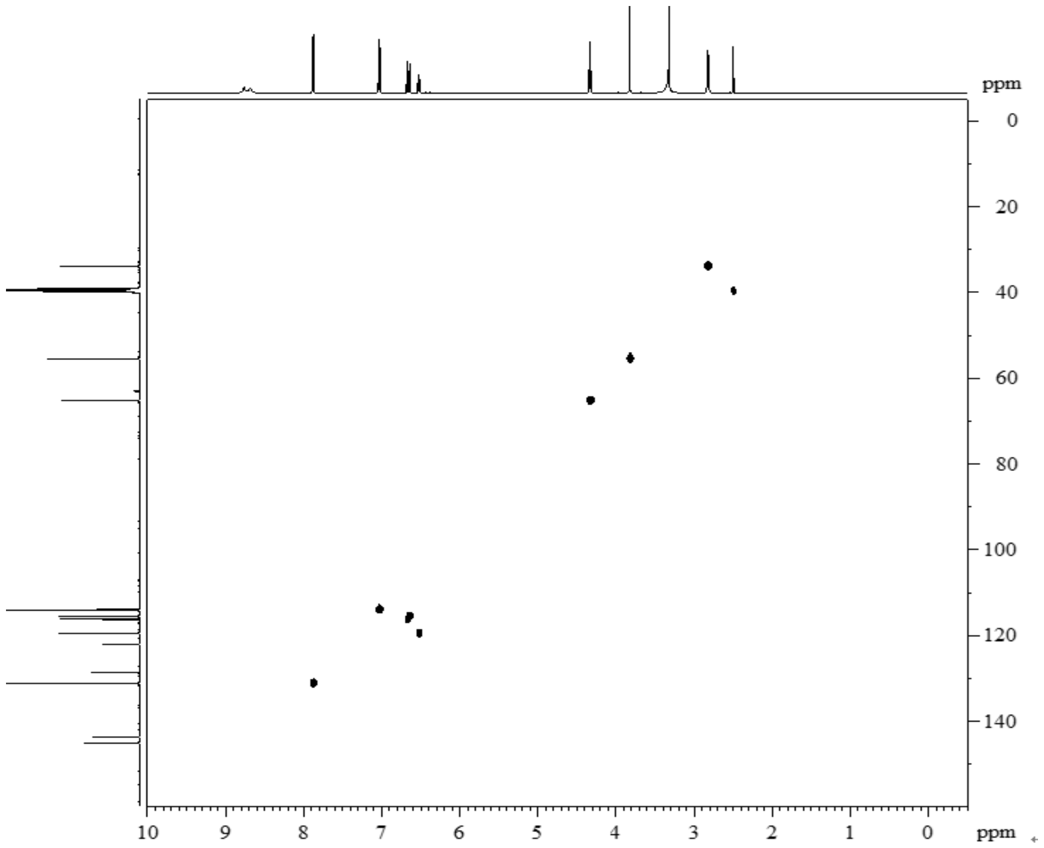

Fig. S5. 2D NMR spectrum (^{1}H-^{13}C HSQC, 500 MHz, DMSO-*d*$_6$) of the 3', 4'-dihydroxyphenethylanisate (1).

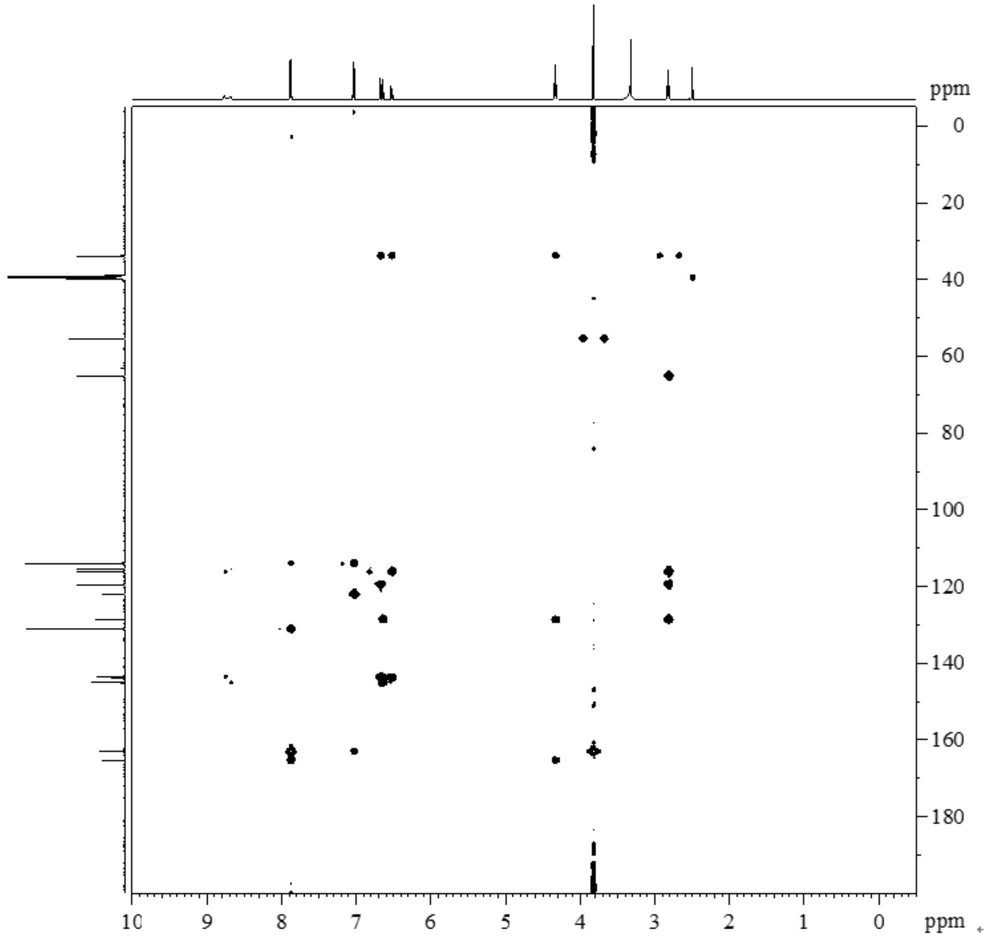

Fig. S6. 2D NMR spectrum (^1H-^{13}C HMBC, 500 MHz, DMSO-d_6) of the 3', 4'-dihydroxyphenethylanisate (1).

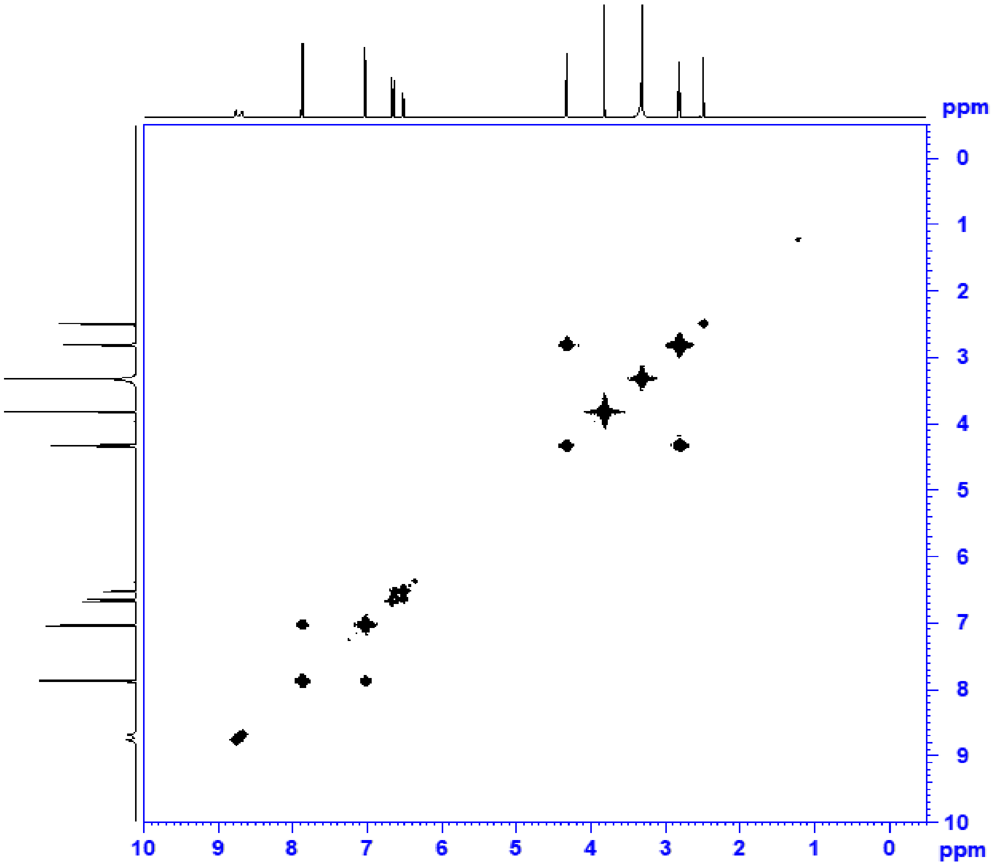

Fig. S7. 2D NMR spectrum (^1H-^1H COSY, 500 MHz, DMSO-*d*$_6$) of the 3', 4'-dihydroxyphenethylanisate (1).

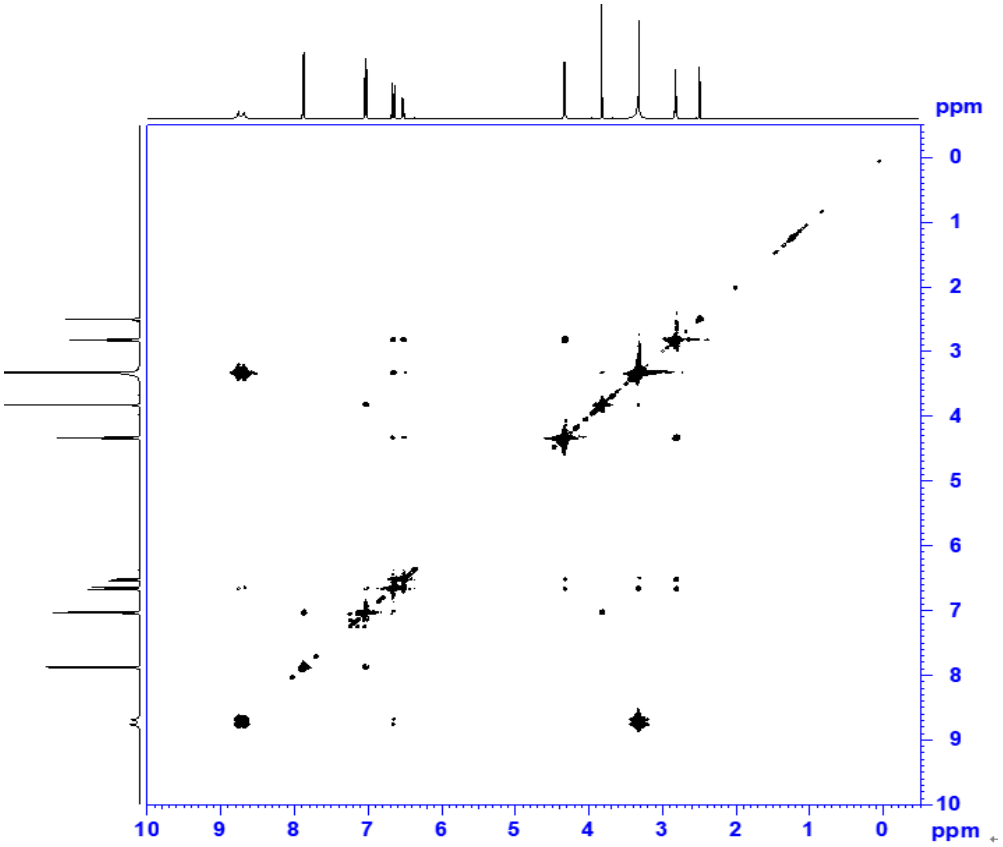

Fig. S8. 2D NMR spectrum (^{1}H-^{1}H NOESY, 500 MHz, DMSO-*d*₆) of the 3', 4'-dihydroxyphenethylanisate (1).

VenomPred 2.0

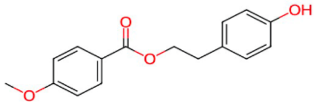

Name	*p*-hydroxyphenethyl anisate

Endpoint	Probability	Endpoint	Probability
Mutagenicity	14 %	Androgenicity	3 %
Carcinogenicity	41 %	Acute Oral toxicity	10 %
Hepatotoxicity	46 %	Skin irritation	10 %
Estrogenicity	51 %	Eye irritation	11 %

The main article describing the web service and its underlying methodologies is: **VenomPred 2.0: A Novel *In Silico* Platform for an Extended and Human Interpretable Toxicological Profiling of Small Molecules.** *J. Chem Inf. Model*. **2023**. https://doi.org/10.1021/acs.jcim.3c00692

Department of Pharmacy

Molecular Modeling & Virtual Screening Laboratory

Fig. S9. *In silico* toxicological predictions of *p*-hydroxyphenethyl anisate (HP) via VenomPred 2.0.

VenomPred 2.0

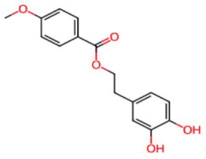

Name	3', 4'-dihydroxyphenethyl anisate

Endpoint	Probability	Endpoint	Probability
Mutagenicity	28 %	Androgenicity	3 %
Carcinogenicity	28 %	Acute Oral toxicity	12 %
Hepatotoxicity	50 %	Skin irritation	9 %
Estrogenicity	54 %	Eye irritation	12 %

The main article describing the web service and its underlying methodologies is: **VenomPred 2.0: A Novel *In Silico* Platform for an Extended and Human Interpretable Toxicological Profiling of Small Molecules.** *J. Chem Inf. Model*. **2023**. https://doi.org/10.1021/acs.jcim.3c00692

Department of Pharmacy

Molecular Modeling & Virtual Screening Laboratory

Fig. S10. *In silico* toxicological predictions of 3', 4'-dihydroxyphenethyl anisate (1) via VenomPred 2.0.
